# Emerging trends in the blood spinal-cord barrier: A bibliometric analysis

**DOI:** 10.1097/MD.0000000000037125

**Published:** 2023-02-02

**Authors:** Bo Xu, Dian Zhang, Bowen Yang, Xin Chen, Zhefeng Jin, Xiaokuan Qin, Guoliang Ma, Kai Sun, Liguo Zhu, Xu Wei, He Yin

**Affiliations:** a Wangjing Hospital, China Academy of Chinese Medical Sciences, Beijing, China; b Graduate School, Beijing University of Chinese Medicine, Beijing, China; c Beijing Key Laboratory of Bone Setting Technology of Traditional Chinese Medicine, Beijing, China.

**Keywords:** Bibliometrics, Blood-Spinal Cord Barrier, Citespace, Spinal Cord Injury

## Abstract

**Background:**

The blood-spinal cord barrier (BSCB) is a unique protective barrier located between the circulatory system and the central nervous system. BSCB plays a vital role in various diseases. However, there is little systematic research and recording in this field by bibliometrics analysis. We aim to visualize this field through bibliometrics to analyze the hotspots and trends of BSCB and in order to facilitate an understanding of future developments in basic and clinical research.

**Methods:**

To conduct a bibliometric study of original publications and their references, the keywords Blood Spinal-Cord Barrier and BSCB are searched and filtered from the Web of Science database (2000–2022), focusing on citations, authors, journals, and countries/regions. Additionally, clustering of the references and co-citation analysis was completed, including a total of 1926 articles and comments.

**Results:**

From the results, 193 authors were identified, among which Sharma Hs played a key role. As far as the analysis result of the clustering of the references is concerned, the most common type in cluster analysis is spinal cord injury (SCI) which is a current and developing research field. The keywords are also the specific content under these clusters. The most influential organization is Univ Calif San Francisco, and “Proceedings of The National Academy of Sciences of The United States of America” magazine is the most cited magazine.

**Conclusion subsections:**

The research on BSCB is booming focusing mainly on “BSCB in SCI” including “activation,” “pathway,” and “drug delivery” which is also the trend of future research.

## 1. Introduction

In recent years, the pathological mechanisms of diseases caused by abnormal barrier function have been widely studied and increasingly valued by the scientific community.^[1]^ Situated at the juncture of the circulatory and central nervous systems, the blood-spinal cord barrier (BSCB) serves as a protective interface for both systems.^[2]^ Molecular movement between blood vessels and spinal cord parenchyma is regulated by the selective permeability of BSCB.^[2]^ As well as non-fenestrated endothelial cells, BSCB consists of the basement membrane, pericytes, and astrocyte terminal processes. BSCB functions as a regulator and protector through the complex tight junction protein network between its components, including occlusive zone 1, occlusal protein, and blocking protein-5.^[3,4]^ As a relatively independent physiological entity, BSCB exhibits some structural and functional differences from the blood–brain barrier (BBB). The main differences are glycogen deposition, permeability, adhesion connexin and transporter molecule,^[2]^ etc. Czech scholar Bartanusz delineated the distinctions between the BSCB and the BBB, emphasizing their conceptual differences.^[2]^ The BSCB differs from the BBB in structure and function. Compared to the BBB, the BSCB exhibits greater permeability and is primarily located near the spinal cord.^[5]^ The integrality of the BSCB has been demonstrated to influence the development or progression of many diseases, including degenerative cervical spondylotic myelopathy,^[6]^ spinal cord injury (SCI),^[5]^ amyotrophic lateral sclerosis (ALS),^[7]^ peripheral nerve injury,^[8]^ spinal cord ischemia-reperfusion injury,^[9]^ and multiple sclerosis (MS),^[10]^ etc.

Although research on BSCB has advanced greatly, there were few studies that provided a comprehensive analysis of the literatures in this field. To establish an international core outcome and knowledge system for the BSCB, this study aims to develop a comprehensive set of challenges, trends, and hotspots for research and clinical studies on BSCB through bibliometric analysis. Introduced by Alan Pritchard in 1969, bibliometrics is defined as “the method of analyzing and quantifying various textual information levels to understand text processing. It also evaluates a discipline’s development nature and trends using mathematical and statistical methods.”^[11]^ Based on the principle of bibliometrics, the present study uses CiteSpace and VOSviewer software to draw knowledge maps. Scholars will be able to gain a quick overview of research hotspots and development trends in the field of BSCB as a result.

## 2. Methods

### 2.1. Data collection

**Figure 1.**
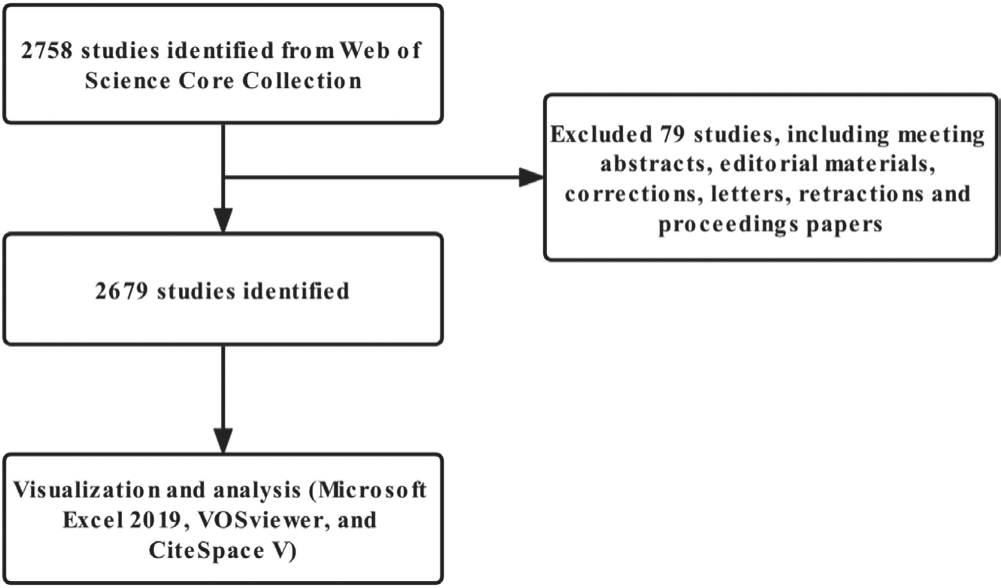
Flowchart of literature selection.

On October 9, 2022, the data were extracted from the Web of Science Core Collection and downloaded in 1 day. The search formula is TS = (Blood Spinal-Cord Barrier) or TS = (BSCB), and the search date is January 1, 2000, to October 9, 2022. There were 2758 articles retrieved, of which 79 articles were excluded, including conference abstracts, news, withdrawn publications, editorial materials, letters, and revisions. In total, 2679 pieces of literature were included and all records and references were exported as plain text files (Fig. 1). Following this, the data were imported into CiteSpace for de-weighting, and all the data were exported uniformly on October 9. As a result, 1926 articles were retrieved.

### 2.2. Data analysis

A visual analysis of the Web of Science data was performed using Microsoft Excel 2019, CiteSpace, and VOSviewer. Based on scientometrics and data visualization, Professor Chen Chaomei developed CiteSpace software for visualizing and analyzing citations.^[12]^ It could present the structure, laws, and distribution of scientific knowledge using data mining, information analysis, and atlas drawings. Additionally, it would be possible to visualize research hotspots and evolution processes intuitively, as well as predict development trends in different fields. Using this method, large amounts of data can be analyzed effectively.^[12–14]^

VOSviewer is scientometrics network analysis software developed by the Center for Science and Technology Research in the Netherlands. It not only offers visual analysis of network data but also generates various maps. A network diagram can be created for academic publications, scientific journals, related authors, research institutions, countries, and keywords. In order to connect the information, there are a number of methods that can be employed, including co-citations, co-occurrences, citations, and bibliographic couplings. In addition to providing density visualizations, VOSviewer software is capable of showing network and overlay visualizations.^[15]^ Clustering of co-occurrences, which indicates their relationship, is a key component of software design. In view of the possibility of a number of correlations arising from variances in intensity and direction, it would be possible to identify different groups based on the clustering of measurement indices of such variances. Though VOSviewer was designed primarily for bibliometric purposes, it can also be used to create virtually any type of web map. Displaying graphics is its most prominent feature, and it is suitable for large-scale data processing.^[12]^

Microsoft Office Excel 2019 was used to analyze the articles. We utilized CiteSpace and VOSviewer software to determine the distribution of countries and regions visually, as well as the number of authors and co-cited authors, the number of journals and co-cited journals, the number of co-cited references, reference cluster analysis, keyword co-occurrences, and timelines.

## 3. Results

### 3.1. The trend of publication outputs

**Figure 2.**
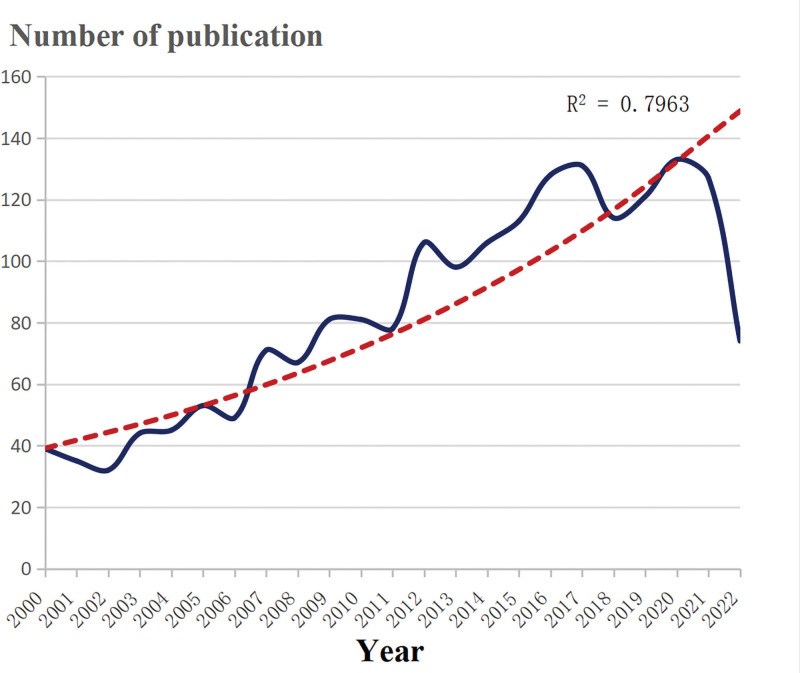
Trends of BSCB publications over the past 23 years. The red dotted line represents the trend line of the number of publications published year by year on the blood-spinal cord barrier. The blue curve represents the changing trend of the number of articles published each year. BSCB = blood-spinal cord barrier.

The number of articles published on BSCB (Fig. 2) showed an overall upward trend between 2000 and 2022. There is, however, an obvious difference between the first 10 years and the later years. During the period of 2000 to 2011, there were fewer than 80 publications each year. Since then, more attention has been drawn to this field and its significant upward trajectory has been noticed. After 2011, over 100 publications have been published each year. In 2020, the number of articles published reached its peak at a record of 133 article publications.

### 3.2. Distribution of countries/regions and institutions

**Figure 3.**
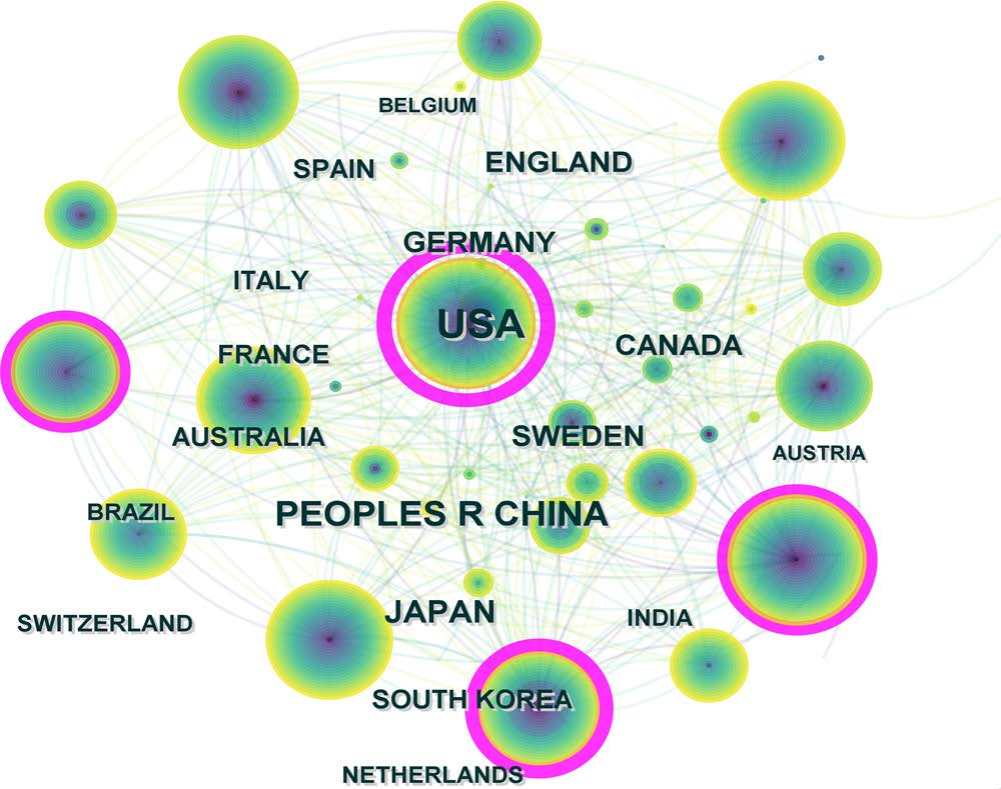
Distribution of publications from different countries/regions. Notes in the Fig represent a country/region. The line between the two represents the connection between them. With more publications, the node’s color gradually changes from purple to yellow. The purple circle outside some nodes means that the node has a strong intermediary centrality.

**Figure 4.**
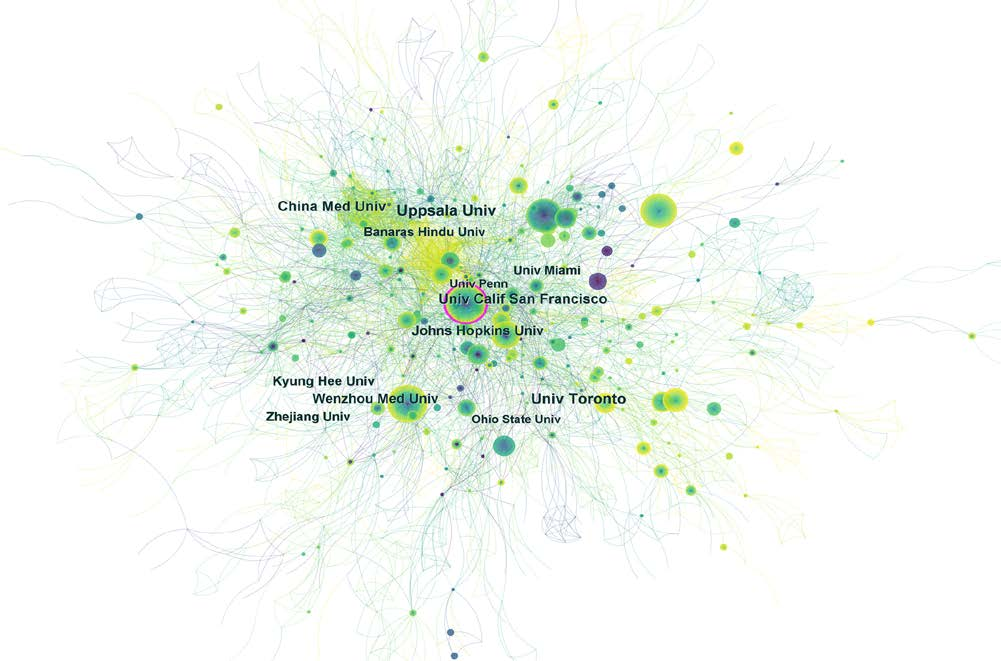
Distribution of publications from different institutions. Notes in the Fig represent an institution. The line between the two nodes represents the connection between the two institutions. With more publications, the node’s color gradually changes from purple to yellow. The purple circle outside some nodes means that the node has a strong intermediary centrality.

Distribution of countries/regions and institutions (Table 1, Figs. 3 and 4) showed that there were a large number of countries and institutions had little or no stable and intensive communication or cooperation. As shown in Figures 3 and 4, USA published the most articles, reaching a record of 759 articles. In addition to the USA, 4 countries, including China, Japan, Germany, Canada, published more than 100 articles. Generally, nodes greater than 0.1 were considered to be relatively significant in terms of centrality, which measures their importance in the overall network. In terms of centralities, USA, England, Germany, and Australia all have centralities greater than 0.1. Due to international collaboration, where two or more countries may publish a single paper, we excluded certain duplicate articles during the data collection phase to ensure accuracy.

**Table 1:** Top 10 countries/regions and institutions related to BSCB.

Rank	Country	Year	Centrality	Count (%)	Institution	Year	Centrality	Count (%)
1	USA	2000	1.09	39.41	Uppsala Univ	2002	0.02	2.60
2	CHINA	2008	0.02	17.29	Univ Toronto	2001	0.02	2.28
3	JAPAN	2008	0.02	6.33	Univ Calif San Francisco	2002	0.11	2.02
4	GERMANY	2008	0.05	5.82	China Med Univ	2008	0.02	1.77
5	CANADA	2008	0.01	5.40	Wenzhou Med Univ	2014	0.03	1.66
6	SWEDEN	2007	0.01	3.84	Johns Hopkins Univ	2001	0.1	1.51
7	SOUTH KOREA	2011	0.00	3.58	Kyung Hee Univ	2012	0.02	1.35
8	ENGLAND	2007	0.05	3.27	Zhejiang Univ	2014	0.02	1.19
9	AUSTRALIA	2008	0.06	2.96	Banaras Hindu Univ	2005	0	1.19
10	FRANCE	2007	0.05	2.91	Univ Miami	2001	0.04	1.09

BSCB = blood-spinal cord barrier.

As shown in Table 1, the publications of Uppsala Univ from Sweden ranked first, with a total of 45 articles, while other institutions also accounted for a certain proportion, such as Univ Toronto, Wenzhou Med Univ, Univ Calif San Francisco, China Med Univ, Kyung Hee Univ, Zhejiang Univ and so on. Among these institutions, Univ Calif San Francisco had the highest centrality.

### 3.3. Authors and co-cited authors

**Figure 5.**
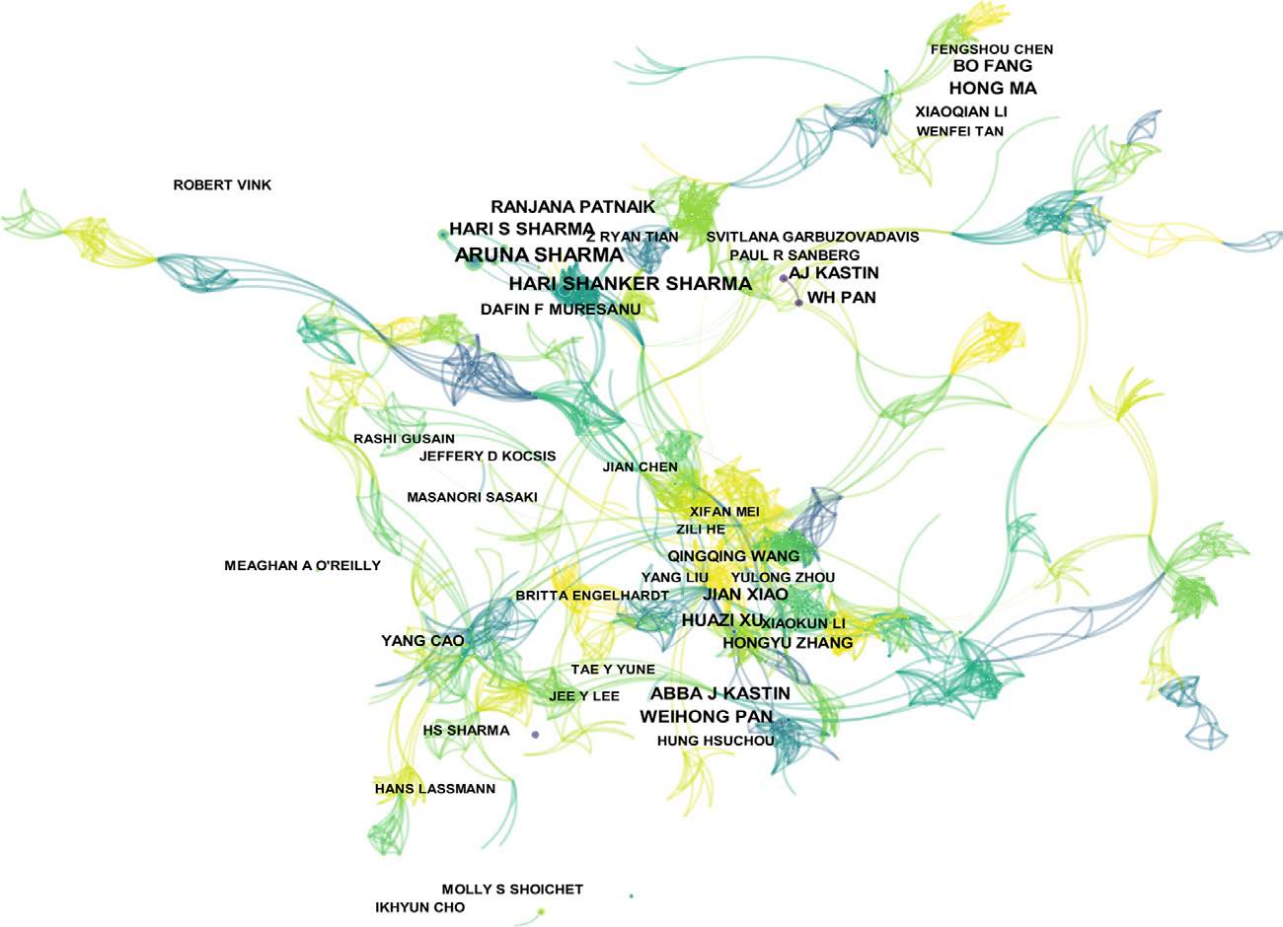
CiteSpace visualization map of authors involved in BSCB Each node represents an author, and the relationship between them is represented by connections. BSCB = blood-spinal cord barrier.

According to statistics, a total of 10,028 authors participated in the publication of articles related to the BSCB. In accordance with Table 2, Aruna Sharma published the largest number of papers (29) followed by Hari Shanker Sharma (24, 1.25%), Hari S Sharma (19, 0.99%), Wei Hongpan (19, 0.99%), Hong Ma (18, 0.93%), and Abba J Kasten (18, 0.93%). As shown in Figure 5, each node represented an author, and the relationship between them was represented by the connections. From the pictures, we can see that certain cooperative network exists among Aruna Sharma, Hari Shanker Sharma, Haris S Harma, and others. Co-citation is defined as a relationship where two or more authors are cited by a subsequent paper at the same time. Of the 1133 cited authors, seven were referenced more than 100 times (Table 2). Abbott Nj was the most frequently referenced person, with a total of 175 citations, followed by Basso Dm (172).

**Table 2:** Top 10 authors and co-cited authors related to BSCB.

Rank	Author	Count (%)	Co-cited author	Citation	Centrality
1	ARUNA SHARMA	1.51	ABBOTT NJ	175	0.12
2	HARI SHANKER SHARMA	1.25	BASSO DM	172	0.12
3	HARI S SHARMA	0.99	SHARMA HS	135	0.11
4	WEIHONG PAN	0.99	NOBLE LJ	124	0.08
5	HONG MA	0.93	POPOVICH PG	121	0.15
6	ABBA J KASTIN	0.93	ZLOKOVIC BV	111	0.1
7	JIAN XIAO	0.88	LEE JY	98	0.03
8	AJ KASTIN	0.88	GARBUZOVA-DAVIS S	96	0.04
9	BO FANG	0.83	ROSENBERG GA	95	0.1
10	RANJANA PATNAIK	0.83	WINKLER EA	91	0.03

BSCB = blood-spinal cord barrier.

### 3.4. Journals and co-cited academic journals

According to the visual analysis of the retrieved literature in VOSviewer, there were 131 journals and 1926 articles published about the BSCB. Among them, “*The Journal of neuroinflammation”* published 60 articles (3.12%), the magazine with the greatest number of related articles, followed by “*Plos One”* with 56 articles (2.91%), and “*Journal of neurotrauma, Experimental Neurology* and *Brain Research”* contributed 56 articles (2.91%), 49 articles (2.54%), and 43 articles (2.23%), respectively (Table 3). In a particular field, the number of co-citations indicates the importance of a journal. In addition, in terms of co-citations (Table 3), *“PNAS”* (1025) was the most frequently cited journal. It was followed by “*Journal of Neuroscience”* (1004), “*Brain Research”* (962), “*Experimental Neurology”* (818), and “*Nature”* (705).

**Table 3:** Top 10 journals and co-cited journals related to BSCB.

Rank	Journal	Count (%)	IFA (2022)	JCR	Co-cited journal	Citation	IF (2022)	JCR
1	journal of neuroinflammation	60 (3.12)	9.587	Q1	P NATL ACAD SCI USA	1025	12.779	Q1
2	plos one	56 (2.91)	3.752	Q2	J NEUROSCI	1004	6.709	Q1
3	journal of neurotrauma	56 (2.91)	4.869	Q2	BRAIN RES	962	3.610	Q3
4	experimental neurology	49 (2.54)	5.620	Q2	EXP NEUROL	818	5.620	Q2
5	brain research	43 (2.23)	3.610	Q3	NATURE	705	69.504	Q1
6	neuroscience	39 (2.02)	3.708	Q3	J NEUROCHEM	676	5.546	Q2
7	molecular neurobiology	37 (1.92)	5.682	Q1	NEUROSCIENCE	660	3.708	Q3
8	journal of neuroimmunology	36 (1.87)	3.221	Q3	BRAIN	619	15.255	Q1
9	journal of neuroscience	35 (1.82)	6.709	Q1	J BIOL CHEM	618	5.486	Q2
10	international journal of molecular sciences	35 (1.82)	6.208	Q1	J NEUROIMMUNOL	614	3.221	Q3

BSCB = blood-spinal cord barrier, IF = impact factor, JCR = journal citation report.

### 3.5. Co-cited references and references burst

**Figure 6.**
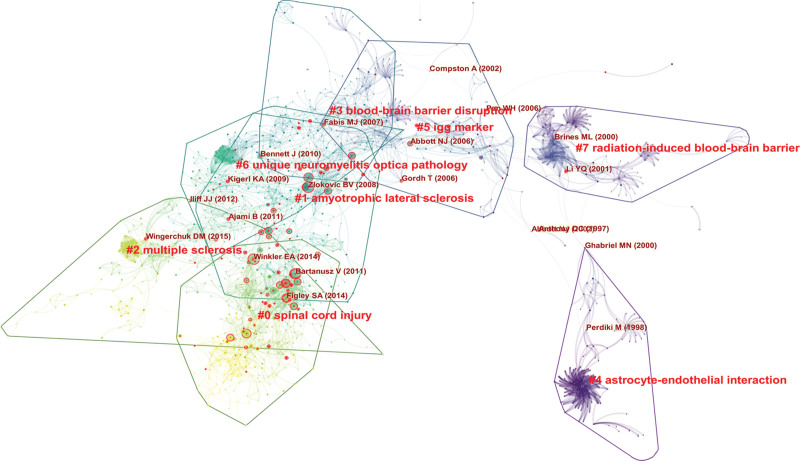
The citation outbreak in the co-citation network. The color in the picture from dark to light represents the time of the published literature from far to near. The red circle outside the dot represents that the article has been referenced more times, and the more the number of references, the thicker the red circle. Citation burst represents that the amount cited in a literature varies greatly in a short period of time. Each cluster represents a collection of citations that describe the same clustering topic.

Whenever 2 references (cited articles) are cited by a third article (cited article), a co-citation relationship has been established. The more co-citations there are, the greater the correlations between the documents. Table 4 shows the 10 most frequently cited articles, of which *Berislav V, et al(2008*)^[16]^ ranked first, followed by *Bartanusz, V., et al (2011*),^[2]^ which should have been considered as the foundation of the field of BSCB. At the same time, by analyzing the burst time of co-citations in the literature, we can get the burst situation and time of the literature. As shown in Figure 6, each node in represented an article. The first co-citation burst of the literature began in 2008, followed by varying degrees of bursts in 2011, 2013, 2014, and 2017, of which the most recent burst was in 2020, and currently most of the cited literatures were being frequently cited indicating that the research on the BSCB may continue to explode.

**Table 4:** Top 10 co-cited references related to BSCB.

Rank	Reference	Citation	Year	Centrality	Ref.
1	The Blood-Brain Barrier in Health and Chronic Neurodegenerative Disorders	30	2008	0	^[16]^
2	The blood–spinal cord barrier: Morphology and Clinical Implications	28	2011	0.01	^[2]^
3	Characterization of Vascular Disruption and Blood–Spinal Cord Barrier Permeability following Traumatic Spinal Cord Injury	28	2014	0.01	^[17]^
4	Blood–spinal cord barrier disruption contributes to early motor-neuron degeneration in ALS-model mice	27	2014	0.01	^[18]^
5	Fluoxetine inhibits matrix metalloprotease activation and prevents disruption of blood–spinal cord barrier after spinal cord injury	25	2012	0.02	^[19]^
6	ALS-causing SOD1 mutants generate vascular changes prior to motor neuron degeneration	24	2008	0	^[20]^
7	Traumatic spinal cord injury	23	2017	0.01	^[21]^
8	Impaired blood–brain and blood–spinal cord barriers in mutant SOD1-linked ALS rat	22	2009	0.04	^[22]^
9	Astrocyte–endothelial interactions at the blood–brain barrier	20	2013	0.03	^[23]^
10	Propitious Therapeutic Modulators to Prevent Blood-Spinal Cord Barrier Disruption in Spinal Cord Injury	20	2017	0	^[24]^

BSCB = blood-spinal cord barrier.

**Figure 7.**
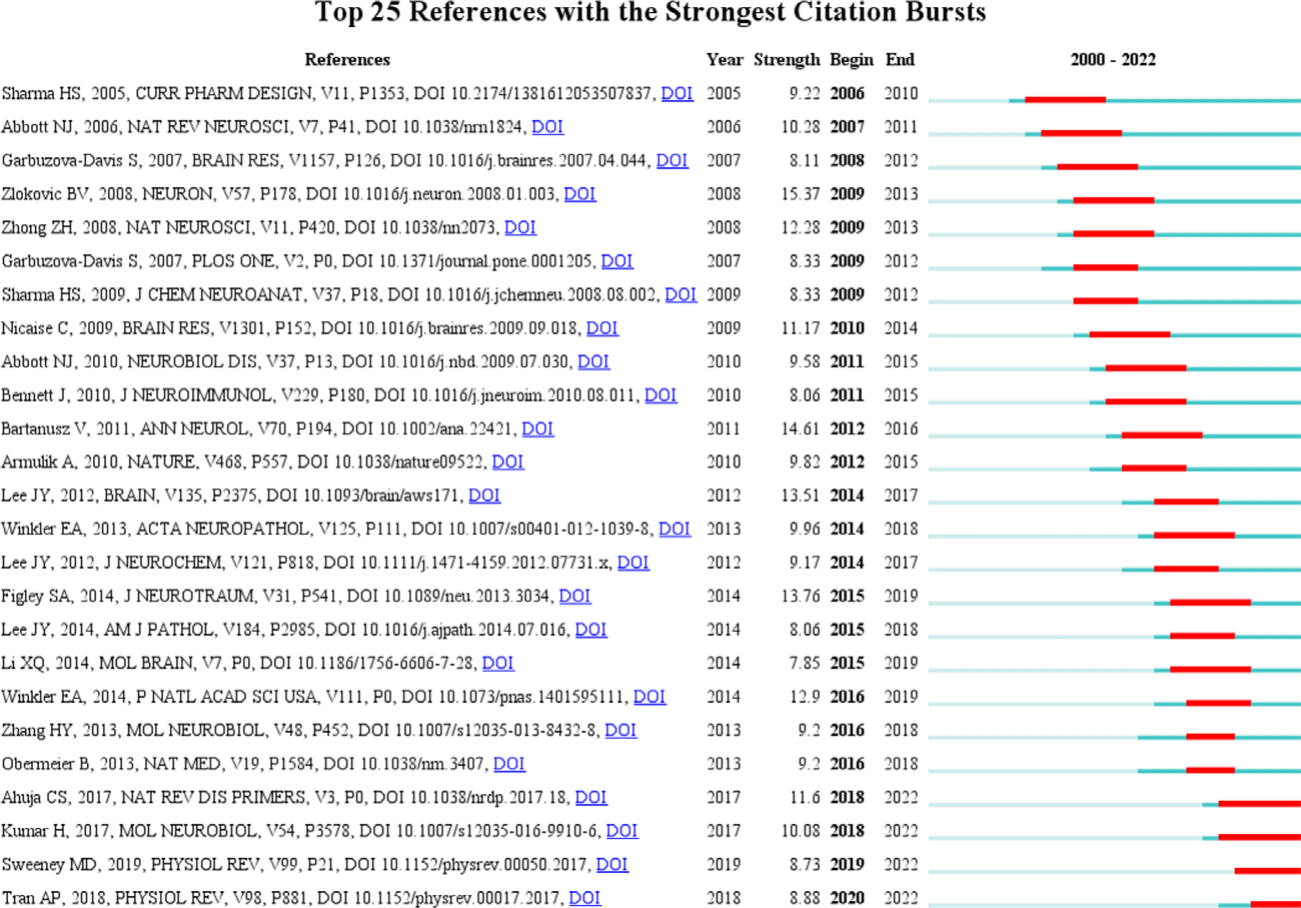
CiteSpace visualization map of top 25 references with the strongest citation bursts involved in BSCB. BSCB = blood-spinal cord barrier.

Figure 7 shows the references with the strongest citation bursts. It should be noted that seven of these articles have a measured strength greater than 10, which indicated that they have a strong influence in this field. The citation burst shown in red nodes in Figure 6 also represented the reference with the strongest citation burst in Figure 7. Most of these red nodes appeared in the largest clusters # 0 spinal cord injury in Figure 6. This also means that SCI is the current key research area in the field of BSCB.

### 3.6. Keywords co-occurrence

Through co-occurrence, we could identify the keywords that were most frequently used. The research words included in this study with a high frequency are listed in Table 5: *blood-brain barrier* (583), *spinal cord* (464), *spinal cord injury* (432), *central nervous system* (353), *multiple sclerosis* (322), *expression* (294), *blood brain barrier* (252), *experimental autoimmune encephalamyelitis* (175) and *rat* (149). It should be pointed out that *the blood-brain barrier, spinal cord*, and *spinal cord injury* appeared more than 400 times. These frequently used research words included in this study may represent the key points in this field.

**Table 5:** Top 10 keywords related to BSCB.

Rank	Keywords	Count	Centrality	Rank	Keywords	Count	Centrality
1	blood brain barrier	583	0.06	11	cell	122	0.07
2	spinal cord	464	0.03	12	brain	121	0.08
3	spinal cord injury	432	0.05	13	blood-spinal cord barrier	120	0.03
4	central nervous system	353	0.02	14	disease	117	0.03
5	multiple sclerosis	322	0.03	15	permeability	117	0.03
6	expression	294	0.04	16	endothelial cell	114	0.03
7	blood-brain barrier	252	0.05	17	model	113	0.04
8	experimental autoimmune encephalomyeliti	175	0.02	18	cerebrospinal fluid	110	0.03
9	rat	149	0.01	19	functional recovery	109	0.03
10	activation	128	0.06	20	inflammation	109	0.02

BSCB = blood-spinal cord barrier.

**Figure 8.**
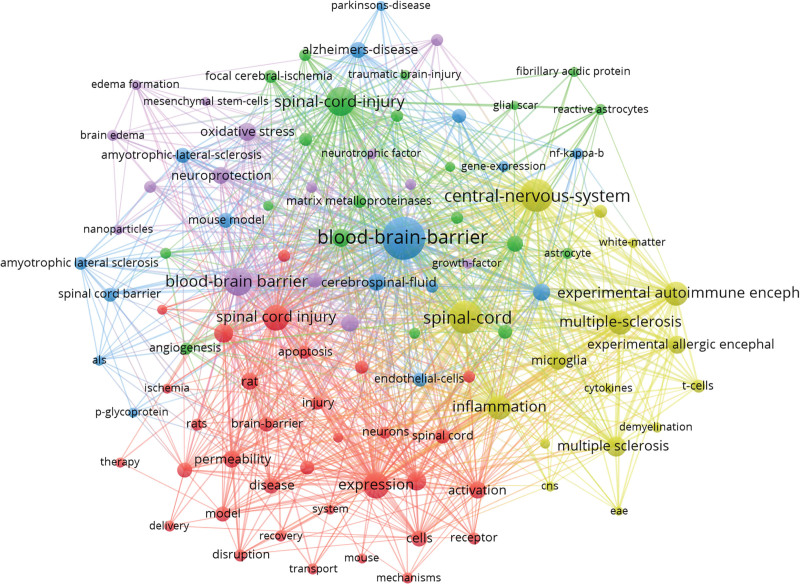
VOSviewer visualization map of keywords clustering analysis related to BSCB. BSCB = blood-spinal cord barrier.

We further conducted a cluster analysis of these keywords based on their co-occurrence. Clustering keywords can provide insight into the relationship within. The keywords identified by the cluster analysis using VOSviewer software were shown in Figure 8. In the Figure 8, the combination of labels and dots formed a unit, and different units formed clusters. A search direction is represented by 5 clusters of red, green, blue, yellow, and purple. Angiogenesis, astrocytes, and SCI were among the keywords included in the orange clustering. Expression, activation, and apoptosis were included in the green clustering. Blue clustering was associated with Alzheimer’s disease, and the BBB while yellow clustering was associated with the central nervous system, experimental autoimmune encephalomyelitis, and MS. The BBB, brain edema, and neuroprotection were the keywords associated with purple clustering. The BSCB has been closely related to diseases such as SCI, Alzheimer’s disease, ALS, and MS over the past few decades.

## 4. Discussion

### 4.1. The trend of publication outputs

The number of published papers on the BSCB showed an obvious upward trend from 2000 to 2022 (Fig. 2). The BSCB was formally proposed by Viktor Bartanusz in 2011 as an independent concept from the BBB,^[2]^ leading to an upsurge in BSCB research. Statistically, the *R*^2^ = 0.7963 indicates that the research enthusiasm in this area is increasing over time, and this trend may continue as time progresses.

### 4.2. General information

As can be seen from (Table 1), the distribution of countries/regions and institutions shows the following that the countries with the largest number of published articles are the United States (759, 39.41%) and China (333, 17.29%), accounting for 56.7% of the total number of published articles. BSCB research is most commonly conducted in the United States and China, based on the data. In the context of a network, centrality refers to the importance of a node within it. A node with a rating greater than 0.1 is generally considered to be of significant importance. The United States ranks as the country with the highest centrality in Table 1. Therefore, it was the most influential among others and played a significant role. As shown in Figures 3 and 4, institutions and countries in terms of their distribution is relatively dispersed. This is the case for the United States and China, the University of Toronto, and Zhejiang University. There is no unified network among these institutions. There was insufficient academic exchange between countries or between research institutes, which indicated that academic exchange was lacking. This situation has hindered the development of this field to some extent. In order to promote the research and development of the BSCB, it is recommended that academic cooperation be strengthened with improved communication and cooperation.

From the perspective of authors and co-citation authors, Aruna Sharma (29,1.51%) has the highest number of published articles. Since authors’ centrality is not particularly prominent, the degree of correlation between authors of the studies on the topic couldn’t be assessed easily, and communication between authors needs to be weighted more. In the terms of co-citation authors, Abbott Nj was the most frequently referenced person. As shown in Figure 7, Abbott Nj’s article had 2 bursts of citation, in 2006 and 2010, respectively. The 2 articles^[23,25]^ discussed about the structure and function of the BBB, and laid the foundation for the following article *Bartanusz, V., et al (2011*),^[2]^ which formally separated BSCB from the field of BBB in 2011.

Six journals were cited more than 700 times (Table 3). Among the top 10 co-cited literatures, 7 are Q2 or above partitions, and 4 of them are Q1 partitions. It is necessary to analyze the distribution sources of kinds of literature in order to gain a better understanding of the situation of key journals in research. The analysis of the publication’s literature sources provided us the information of the current situation of the core journals in the field. Data indicated that most of the cited literatures were from journals with high impact factors, suggesting that the research on the BSCB is of high academic value globally.

### 4.3. Hotspots and emerging trend in BSCB

**Figure 9.**
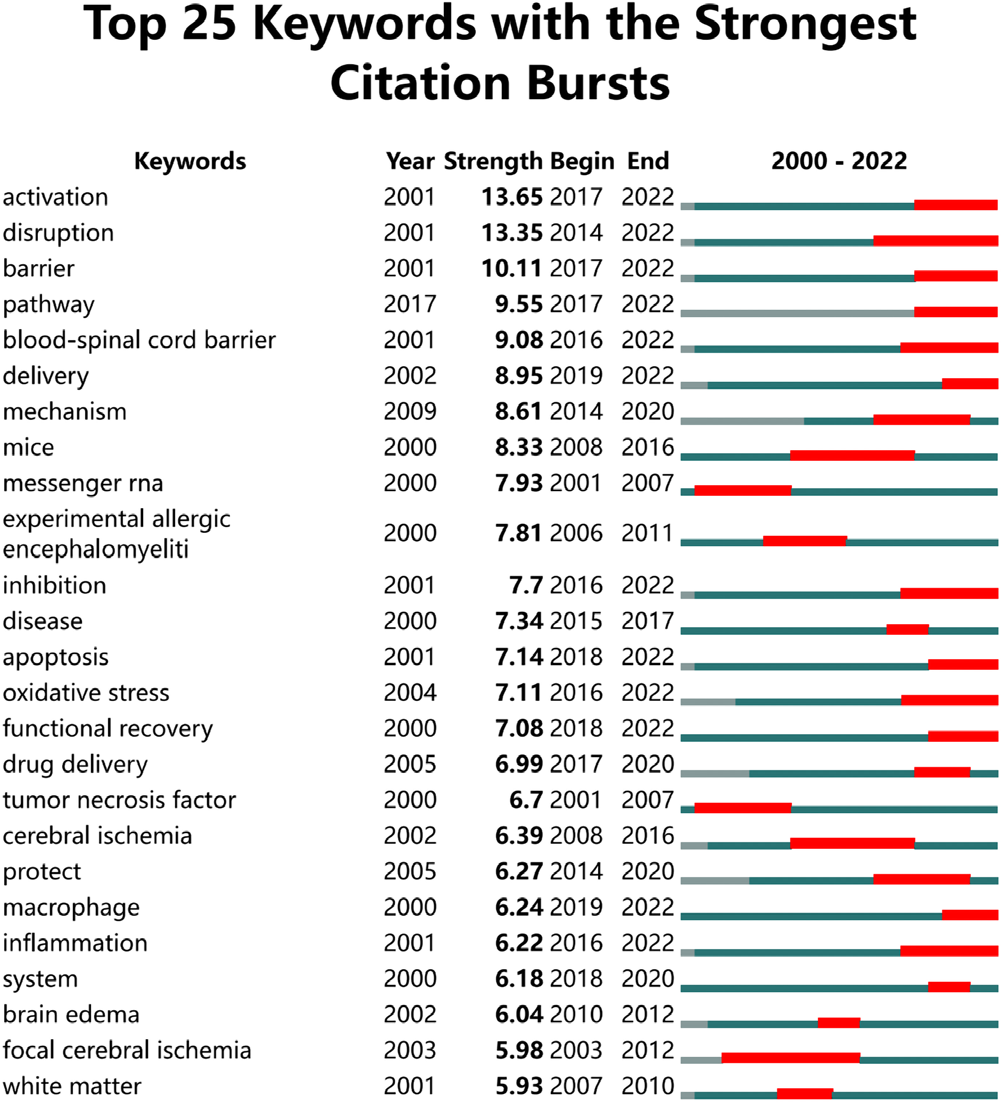
CiteSpace visualization map of timeline viewer related to BSCB. BSCB = blood-spinal cord barrier.

Through co-occurrence analysis of keywords, which usually are the focus and the core content a literature, research hot spots can be assessed in terms of their distribution and development in a particular field. As shown in Table 5, the keywords with high frequency were blood–brain barrier (583), spinal cord (464), spinal cord injury (432), central nervous system (353) and multiple sclerosis (322), expression (294), blood–brain barrier (252) and the experimental autoimmune encephalomyelitis (175). As indicated by the frequency, “BBB” ranked the first in the co-occurrence analysis of keywords, rather than “BSCB.” As a different concept from BBB, BSCB first appeared in 2011 and have appeared 120 times until now (Fig. 9). Therefore, it is reasonable to suggest that BSCB is an emerging domain. Furthermore, citation burst on certain references and keywords, are also prevalent ways to reflect the research hot spots and emerging trend. The landmark reference with the strongest citation burst is Berislav V, et al (2008),^[16]^ followed by Bartanusz V, et al (2011),^[2]^ which demonstrated that the BSCB is an extension of the BBB, and may provide a new research area for the diseases. The cluster analysis based on the keywords finally formed the cluster of 5 colors (Fig. 8). These clusters were almost all related to diseases. This showed that the role of BSCB is closely related to certain specific diseases.

**Figure 10.**
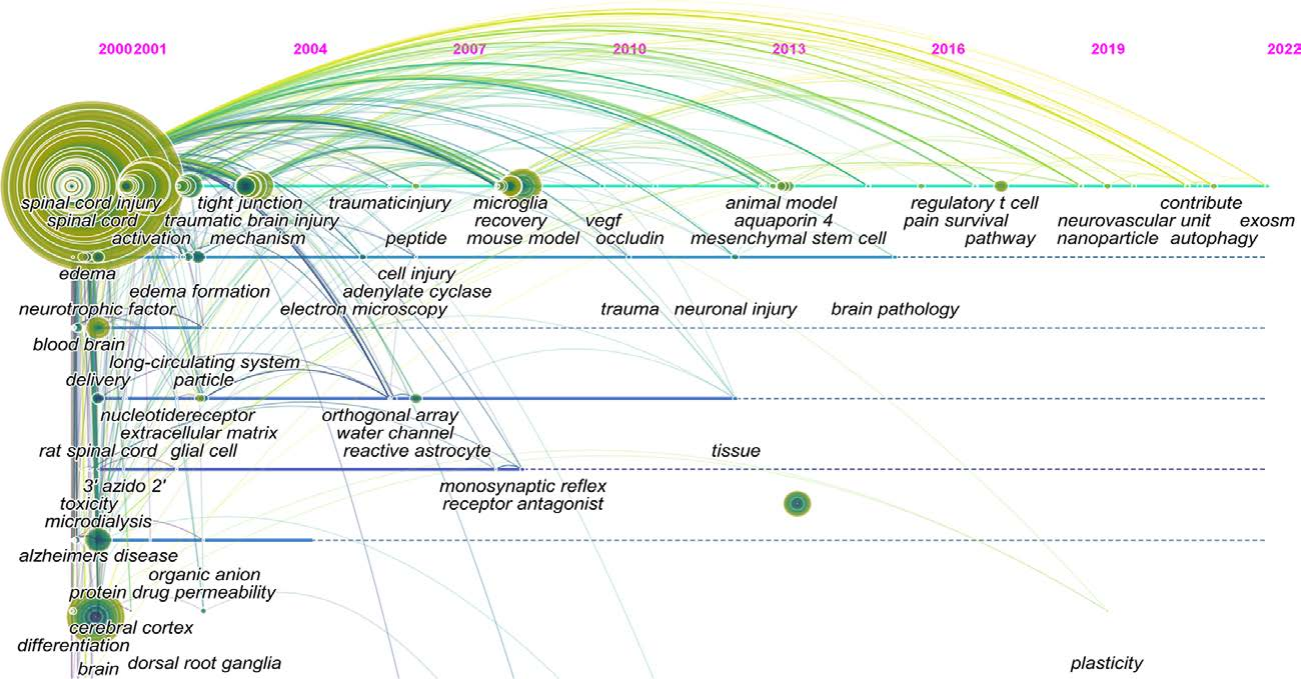
CiteSpace visualization map of top 25 keywords with the strongest citation bursts involved in BSCB. BSCB = blood-spinal cord barrier.

MS, SCI, and ALS are the top 3 diseases of special interest in BSCB. However, other than those 3 top choices, there were sufficient evidence to demonstrate that “SCI” was a topic of most interest in BSCB. Firstly, in the field of BSCB, “spinal cord injury” appears to be the leading disease in keyword co-occurrence analysis (Table 5). In addition, most articles with top 10 citations were about spinal cord injuries (Table 4). As mentioned before, citation burst is a representation of emerging trend. There is a recent co-citation cluster with concentrated citation burst, labeled as “#0 spinal cord injury” (Fig. 6). Therefore, this domain should be considered as the current hot spot in BSCB, consistent with the result from co-occurrence analysis of keywords. Furthermore, citation bursts of keywords indicated that activation, disruptions, pathways, apoptosis, and delivery had attracted the most attention in BSCB (Fig. 10), which could be a good direction to explore the relations between BSCB and spinal cord injuries.

### 4.4. BSCB in SCI

As discussed before, SCI is a hot spot in BSCB. In 2005, Sharma^[26]^ preliminary proved that BSCB plays an important role in the regulation of spinal cord microenvironment. Then Figley et al’s 2014 paper^[17]^ used Evans Blue to reveal the characterization of BSCB permeability after SCI. After SCI, a series of pathological changes could occur. Following the initial physical injury, with BSCB destroyed, and hemorrhage and ischemia occurring, a large number of cell death, edema, and inflammation could be next. Apoptosis of neurons and glial cells continues to expand on the initial basis, and finally fades into a cavity surrounded by glial cells/ fibrotic scars. Regarding the series of changes after SCI, the correlation between BSCB and SCI has been further studied.

It was found that the keyword *activation* had the strongest citation burst since 2017 (Fig. 10), which was also the start time of the citation burst on 2 references, describing BSCB disruptions and protein activation following SCI.^[21,24]^ During this period, investigation of the major proteins that mediated BSCB destruction and promoted BSCB repair after SCI had become a hot spot. As part of the healing process following spinal cord injury, matrix metalloproteinases (MMPs) are involved; tumor necrosis factor is another, as is heme oxygenase-1, angiopoietin, bradykinin, nitric oxide, and endothelin (ET).^[24]^ As a result of the SCI, matrix metalloproteinases are activated, destroying the BSCB, resulting in an infiltration of blood cells, inflammation, and neuronal death, which causes permanent neurological damage.^[19]^ A metalloproteinase is an extracellular zinc- and calcium-dependent endopeptidase responsible for the degradation of extracellular matrix and other extracellular proteins (MMPs).^[27]^ The excessive proteolytic activity of MMPs is harmful and can lead to many pathological conditions.^[28–31]^ In 2014, Lee et al^[32]^ confirmed that MMP-3 played a role in promoting early BSCB destruction and bleeding in SCI and could impede long-term neurological recovery after SCI. MMP-9 could lead to abnormal vascular permeability after SCI.^[33]^ In recent years, BSCB damage and secondary SCI have been effectively reduced by inhibiting the activity of MMP protein.^[34,35]^ After SCI, overactivated ETs is harmful.^[36–38]^ The ETs are the most potent vasoconstrictors known and are vital for the development of embryos, the remodeling of vascular structures, and the healing of wounds.^[39,40]^ Recent research showed that ETs could induce ischemia and oxidative stress.^[41–43]^ Apoptosis of glial cells after ischemia inevitably leads to the influx of tumor necrosis factor-α and nitric oxide in the peripheral circulation, which could aggravate the inflammatory reaction and promotes SCI.^[9,44]^ Heme oxygenase-1 can promote the programmed death of injured cells through the inherent suicide procedure, thus protecting the cells from further damages.^[45]^ In 2013, Zhang et al’s^[46]^ team revealed that SCI is related to excessive cellular autophagy and ubiquitin accumulation in terms of regulating autophagy and ubiquitin accumulation. At the same time, it is also proved that basic fibroblast growth factor can inhibit excessive autophagy and enhance the clearance of ubiquitin proteins by activating the PI3K/Akt/mTOR signal pathway, indicating a new trend in drug development for the central nervous system injury.^[46]^

The keywords *pathway* was found to have a strong citation burst since 2017 as well. For the past few years, several pathways have been found to regulate the BSCB effectively. The study of some pathways involved in the regulation of BSCB clarified the influence/causal relationship between BSCB damage and SCI. For example, Jiang et al^[47]^ and his colleagues demonstrated that down-regulation of ubiquitin-specific protease 4 (USP4) promoted the activation of microglia and subsequent neuronal inflammation after SCI via NF-κB pathway. By inhibiting this pathway, further regulation of BSCB can also reduce secondary SCI. There is also much research interested in investigating the effect of certain components in the process of protecting BSCB’s integrity. Zhao et al^[48]^ revealed that the SIRT1/AMPK signaling pathway is mediated by resveratrol, which protects against SCI. Chen et al^[49]^ demonstrated that Valproic acid reduces the inflammation caused by traumatic SCI via the STAT1 and NF-B pathways dependent on HDAC3. It is still necessary to conduct more experiments to explore the mechanism of drugs alleviating secondary injury of BSCB after SCI.^[50–52]^

Drug delivery through BSCB has been attracting more and more attention.^[53–55]^ This keyword also showed strong citation burst in recent year (Fig. 10). In the past few years, BSCB has developed a comprehensive drug delivery system aimed at specifically delivering therapeutic drugs to the site of injury. Among those delivery systems, nanoparticles have become the mainstream choices in drug delivery systems due to their small size, strong encapsulation abilities, long drug release duration, and excellent biocompatibility.^[56]^ In particular, exosomes, a substance derived from organisms, is able to increase motor function and reduce BSCB leakage, thereby reducing secondary SCI.^[57–59]^ Other types of nanoparticles, including metals, polymers, lipids, etc., also showed great potential as tracers and drug delivery systems in SCI.^[60–63]^ BSCB has drawn much attention in SCI, but further research is needed to confirm its significance in the future.

## 5. Limitation

CiteSpace and VOSviewr both have their limitations. Bibliometric analysis was not possible with some other databases other than the Web of Science Core Collection. The literature we obtained was published between 2000 and 2022, but as time pass by and with the Web of Science Core Collection continually updated, the results included in this study during that timeframe may differ from the actual number of types of literature included in this study. Furthermore, the quality of the articles included in this study is inherently uneven, which may undermine its credibility. As a final point, several of the principal keywords in the paper have not been fully analyzed, and this may have a detrimental effect on the results. However, there is no doubt that the visual analysis of the literature based on CiteSpace and VOSviewr software can provide scholars with research hotspots, development trends, and research directions in the field of BSCB, enabling them to grasp the foremost advance in the field and lay the foundation for their related research as quick as possible.

## 6. Conclusion

There is considerable research value in the physiological study of the BSCB, and the application of the BSCB to diseases has a wide scope of application prospects. A visual analysis of CiteSpace and VOSviewer software indicates an increase in interest in BSCB every year, based on CiteSpace and VOSviewer software. Globally, the United States and China are the leading countries in this study. It is the University of California San Francisco that has the greatest impact on research results, while countries and institutions need to strengthen their cooperation and communication. Abbott Nj has made an outstanding contribution to the field of BSCB research. Articles about BSCB are frequently cited in journals with international influence, which indicates that BSCB has received wide attention globally. Currently, research on BSCB focuses primarily on BSCB in SCI including “activation,” “pathway,” “drug delivery,” which will also be a focus of future research.

## Acknowledgments

The authors thank Wangjing Hospital, China Academy of Chinese Medical Sciences, for their support of this work and the reviewers for allowing us to make improvements to the manuscript.

## Author contributions

**Conceptualization:** Bo Xu.

**Data curation:** Dian Zhang, Bowen Yang, Guoliang Ma.

**Formal analysis:** Bo Xu, Xin Chen, Zhefeng Jin, Kai Sun.

**Methodology:** Bowen Yang, Xiaokuan Qin, He Yin.

**Writing – original draft:** Bo Xu, Dian Zhang, Bowen Yang.

**Writing – review & editing:** Liguo Zhu, Xu Wei, He Yin.
